# A method for transplantation of human HSCs into zebrafish, to replace humanised murine transplantation models

**DOI:** 10.12688/f1000research.14507.2

**Published:** 2018-12-23

**Authors:** Noémie Hamilton, Ian Sabroe, Stephen A. Renshaw

**Affiliations:** 1 The Bateson Centre, University of Sheffield, Sheffield, S10 2PT, UK; 2 Department of Infection, Immunity and Cardiovascular Disease, University of Sheffield, Sheffield, S10 2PT, UK

**Keywords:** zebrafish, stem cell transplantation, xenograft, humanised zebrafish

## Abstract

Haematopoietic stem cell (HSC) transplantation is a critical therapy for haematopoietic malignancies and immune disorders. Incomplete or delayed engraftment of HSCs in the host results in increased risk of infection and morbidity. The mechanisms of HSC engraftment are poorly understood and understanding these processes will increase transplantation success on many levels.

Current animal models are immunocompromised 'humanised' mice transplanted with human HSCs. Harmful procedures include genetic manipulations and irradiation to ablate the mouse immune system, and opaque mouse tissues make visualisation of the early steps of HSC engraftment impossible. There is a need for new models to offer alternatives to humanised mice in the study of HSC transplantation.

Here we described a detailed method for transplantation of human HSCs into zebrafish, before the onset of adaptive immunity. Human HSCs were purified from whole blood by enrichment of the CD34 cell population using a positive magnetic selection and further purified using an anti-CD34 antibody and cell sorting. Sorted CD34 cells were transplanted into the blood stream of 52 hour old zebrafish larvae. Human HSCs home into the zebrafish haematopoietic niche, where they engage with endothelial cells and undergo cell division. Our model offers the opportunities to image *in vivo* human HSC engraftment in a transparent organism, without the myeloablative strategies used in mice, and provides a unique system to understand the dynamic process of engraftment and replace current murine models.

This technique can be applied to current engraftment protocols to validate the viability and efficiency of cryofrozen HSC grafts. This humanised zebrafish model will be instrumental to develop the 3Rs values in stem cell transplantation research and our detailed protocol will increase the chances of uptake of this zebrafish model by the mouse community.

Research highlights **Scientific benefits:**

- Zebrafish embryos are transparent and represent a tractable system for imaging Zebrafish do not develop an adaptive immunity for the first 2 weeks of life, providing a large time window to perform xenotransplantation

**3Rs benefits:**

- Zebrafish larvae can be used to replace mouse models in stem cell transplantation research
- Use of zebrafish larvae also avoids subjecting mice to severe irradiation procedures and eliminates the risk of contracting fatal infections

**Practical benefits:**

- Zebrafish are cheaper to raise and host in fish aquariums that can hold thousands of animals
- A pair of Zebrafish produces hundreds of small embryos easily transplanted therefore offering the opportunity to perform high-throughput experiments

**Current applications:**

- Studying the engraftment mechanism of human HSCs
- Drug screens to identify new drugs to improve engraftment rate of HSCs
- Identifying new human HSC markers to improve the rate and speed of engraftment

**Potential applications:**

- Assessing the viability and efficacy of human HSC grafts before transplanting into patient
- Replace the use of mouse models during the optimisation phase of HSC transplantation protocols

## Introduction

Transplantation of healthy haematopoietic stem cells (HSCs) is a critical therapy for a wide range of malignant haematological and non-malignant disorders and immune dysfunction ( Snowden *et al.*, 2012; Sykes & Nikolic, 2005; Thomas *et al.*, 1957). In successful stem cell transplantation (SCT), immune reconstitution following ablation of native immunity leads to the recovery of immune function. Healthy transplanted stem cells home to haematopoietic niches in the host and differentiate into multi-lineage blood cells, providing the patient with a new immune system ( Sullivan *et al.*, 2010). Around 2000 people in the UK are in need of SCT every year, and as more hospitals are performing this high-risk life-saving procedure, there is a growing need in improving current protocols. ( http://www.anthonynolan.org)

HSCs are collected from: 1) blood harvested from peripheral blood by apheresis following mobilisation by G-CSF cytokine treatment; 2) umbilical cord blood; or 3) bone marrow from donors or patients. HSCs are enriched post-collection by positive selection for the CD34 stem cell marker. Conditioning or myeloablation of the host bone marrow by chemotherapy is necessary to ablate malignant or autoreactive immune populations, adding a considerable risk of infection while engraftment occurs. Incomplete or delayed engraftment results in delayed immune system recovery, increasing considerably the risk of infection and associated morbidity and mortality. The regulating mechanisms of the homing and migration steps of HSC engraftment are poorly understood and understanding these processes will increase transplantation success on many levels. Accelerated and more complete engraftment will reduce morbidity and mortality associated with transplantation, both during engraftment and long-term immune recovery.

The only models currently employed to study human HSC engraftment are immunocompromised mice transplanted with human HSCs, also called 'humanised' mice ( Tanner *et al.*, 2014). Although these mouse models informed current stem cell transplantation protocols, they involve prolonged harmful procedures and it remains difficult to assess and visualise the early steps of engraftment due to the opacity of their tissues. Multiple mouse strains have been generated to create suitable immunocompromised hosts to allow engraftment of a fully developed adaptive immune system ( Tanner *et al.*, 2014). In most studies, additional harmful irradiation regimes are used to prevent early rejection of the transplant by the immune system. These immunodepleted mice must be grown to adulthood in order to assess engraftment success. They live their entire lives undergoing severe procedures with high maintenance requirements, since they need to be homed in sterile rooms and fed sterile food to avoid fatal infection due to defective immunity. Published articles test multiple conditions on groups of 5 to 6 mice sacrificed at various time points, resulting in an average of 40 mice per publication. In 2015, Pubmed searches yielded 25 publications using immunocompromised mice for HSC transplant studies, representing around 1000 mice each year worldwide – all undergoing severe procedures over a long period of time. As the demand for stem cell transplantation therapy increases, more efficient and less dangerous procedures will be demanded, which will require an even higher mouse usage to optimise current protocols.

Zebrafish are already established as a successful model to study the haematopoietic system, with significant homology with mammals ( de Jong & Zon, 2005; Gering & Patient, 2005; Kissa & Herbomel, 2010; Renshaw & Trede, 2012; Traver *et al.*, 2003; White *et al.*, 2008). Imaging of zebrafish transparent embryos remains a powerful tool and has been critical to confirm that the zebrafish Caudal Haematopoietic Tissue (CHT) is comparable to the mammalian foetal haematopoietic niche ( Gering & Patient, 2005; Kissa & Herbomel, 2010; Tamplin *et al.*, 2015). Xenotransplantation in zebrafish embryos has revealed highly conserved mechanisms between zebrafish and mammals. Recently, murine bone marrow cells were successfully transplanted into zebrafish embryos, revealing highly conserved mechanism of haematopoiesis between zebrafish and mammals ( Parada-Kusz *et al.*, 2017). Additionally, CD34 enriched human cells transplanted into zebrafish were shown to home to the CHT and respond to zebrafish stromal-cell derived factors ( Staal *et al.*, 2016).

We propose that transplanting human HSCs into zebrafish larvae, before the onset of adaptive immunity, will offer unprecedented *in vivo* opportunities to understand stem cell engraftment and help to shift current research towards a 3Rs approach to reduce and refine, and finally replace the usage of mice in HSC transplant studies. Here we describe a detailed transplantation protocol of pure human HSCs into zebrafish larvae. Human PBMCs were enriched for CD34 cells and further purified by cell sorting using the HSC marker CD34. Transplantation of human HSCs into 52hpf larvae was achieved by injection into the Duct of Cuvier. We have evidence that human HSCs home to the zebrafish CHT, where they interact with endothelial cells and undergo cell division. This conserved engraftment mechanism makes zebrafish a unique model to study HSC engraftment and we wish to highlight the significant opportunities to impact on reductions in mammalian model usage. This could lead to new clinical applications to improve the speed and extent of human HSC engraftment.

Humanised zebrafish could offer a welfare improvement compared to current mouse models, as early zebrafish larvae do not require immunodepletion by irradiation or multiple genetic modifications to avoid graft rejection. Zebrafish do not develop functional adaptive immunity until 2 weeks of age and therefore do not require severe procedures if the transplantation occurs in this time window ( Langenau *et al.*, 2004). Using a model with substantially reduced risk of fatal infection and eliminating the need for irradiation significantly refines the current substantial severity protocols.

Additionally, upon transplantation of human stem cells, mice must be grown for several months to assess engraftment success by analysing the reconstitution of the immune system. The transparency of the zebrafish larvae offers a unique system allowing direct live imaging of transplanted cells to visualise cell behaviour and interactions. This will allow the selection of only successful engrafted animals for further analysis and will therefore improve experimental design and throughput whilst simultaneously reducing animal numbers.

Moreover, humanised mice are still being used to optimise protocols of HSCs transplantation, such as source, type and number of cells transplanted and testing different expansion protocol ( Tanner *et al.*, 2014). High-throughput assays can easily be performed using zebrafish larvae, with the scientific advantage of generating a broader range of outputs at lower cost. This, combined with transparency of the larvae to quickly assess engraftment, we expect that our model may be used to replace the use of mouse models during these optimisation phases. Mammalian models could then be reserved purely for the analysis required by law before clinical trials are performed. This could replace all the murine models currently used to optimise protocols with zebrafish larvae before the onset of independent feeding – considered in law and ethics as a model of significantly lower neurophysiological sensitivity.

Finally, humanised mice used to study human stem cell transplantation require an average of 1×10 ^5^ CD34+ cells per animal. 100ml of blood yields approximately 1×10 ^8^ peripheral blood mononuclear cells (PBMCs) from which 0.1%, or 1×10 ^5^ CD34+ cells are routinely isolated, enough for just one mouse. However, we have demonstrated that zebrafish larvae only need 20 to 50 cells transplanted to successfully engraft. Thus, replacing mouse models by a smaller non-protected vertebrate will allow testing of multiple conditions into multiple animals from the same human donor. This will decrease natural variations therefore improving reproducibility and reliability of research performed in this field.

## Methods

A detailed protocol of the procedure is available in Supplementary File 1.

### Zebrafish husbandry

Zebrafish ( *Danio Rerio*) were raised and maintained under the Animal [Scientific Procedures] Act 1986 (Home Office Project Licence 70/8178 used to raise and maintain transgenic lines) using standard protocols ( Nüsslein-Volhard & Dham, 2002). Zebrafish adults were hosted in UK Home Office-approved aquaria at the Bateson Centre, University of Sheffield, and kept under a 14/10 light/dark regime at 28 degrees. The endothelial cells transgenic reporter line *Tg(kdrl:HRAS-mCherry-CAAX) (allele code s916)* ( Chi *et al.*, 2008) is referred to as *kdrl*:mCherry in the manuscript.

### Human PBMCs collection and HSCs enrichment

Blood was taken from healthy volunteers (pool of 9 donors, males and females, aged between 18 and 40) with written informed consent and ethical approval from the South Sheffield Research Ethics Committee (STH18729). Human PBMCs were collected after routine neutrophil preparation by dextran sedimentation followed by plasma-Percoll gradient centrifugation from whole blood ( Prince *et al.*, 2017). PBMCs were further purified by positive selection using the CD34 MicroBead kit (Miltenyl Biotech, Bergisch Gladbach, Germany). Beads were not removed as they are expected to detach within 24h of labelling.

### CD34 cell sorting of Human HSCs

CD34 enriched PBMCs were labelled with anti-Human CD34-eFluor450 antibody (eBioscience- RRID:AB_10734946) and positive cells were sorted using Fluorescence Activated Cell Sorting (FACS). Sorted cells were labelled with Fluorescein and injected into the Duct of Cuvier in 52hpf zebrafish.

### Microscopy

Zebrafish larvae were sedated in Tricaine and embedded in 0.8% low melting point agarose. High-resolution imaging was performed using a Spinning Disk confocal microscope.

### Statistical analysis

Sample size (n=10) is represented by number of larvae used to count human HSCs present in the CHT or within perivascular pockets. Paired T-test and Pearson correlation were used to assess significance using free GraphPad software online.

## Results

### Human CD34 cells can be purified from human whole blood

**Figure 1.**
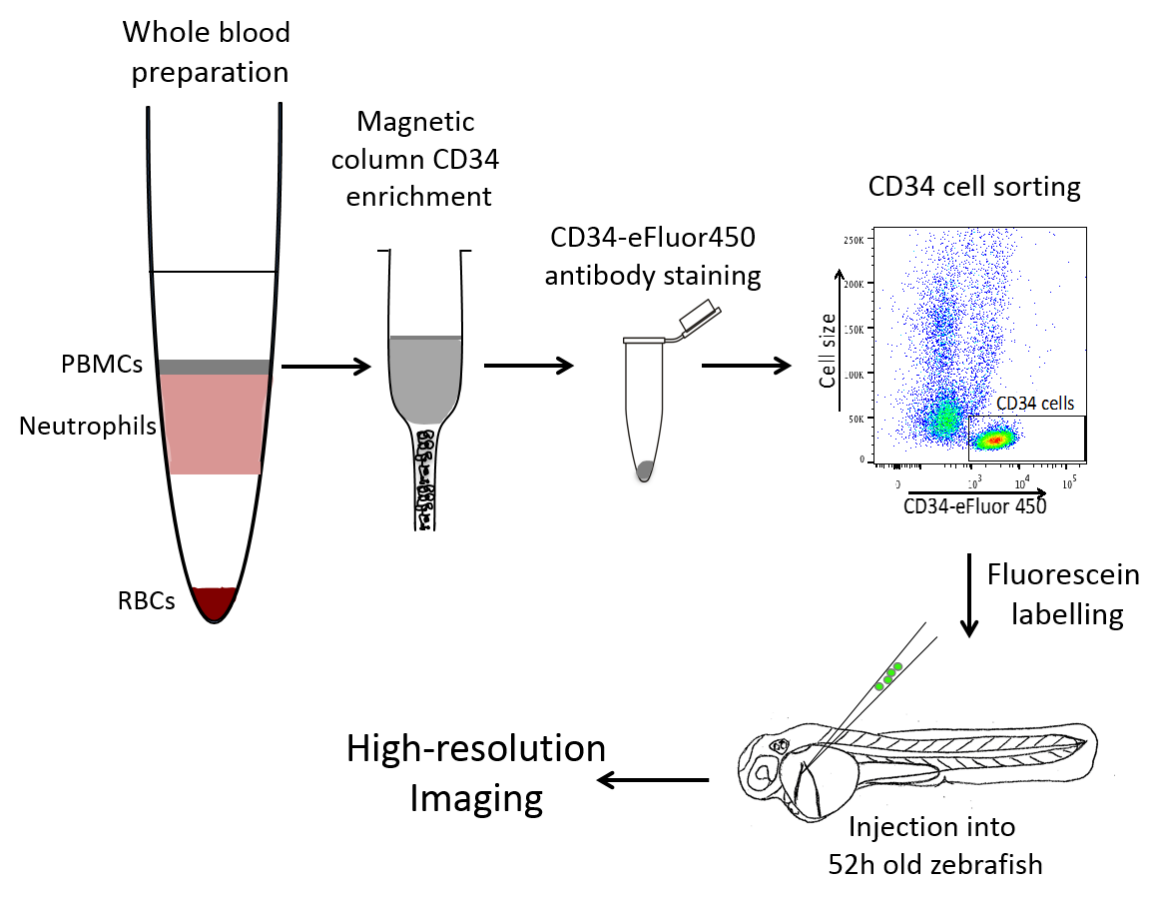
Diagram of our protocol: How to purify human CD34 cells from whole blood and transplant into zebrafish. Whole blood preparation by Percoll gradient allowed us to separate peripheral blood mononuclear cells (PBMCs) from neutrophils and red blood cells (RBCs). PBMCs were enriched for CD34 cells using a positive selection magnetic column. A pure CD34 cell population was sorted using a human anti-CD34eFLuor450 antibody by FACS. CD34 cells were labelled using fluorescein and injected into the Duct of Cuvier of 52 hour post fertilisation zebrafish larvae. Animal with human cells in their CHT were selected for further high-resolution imaging.

**Figure 2.**
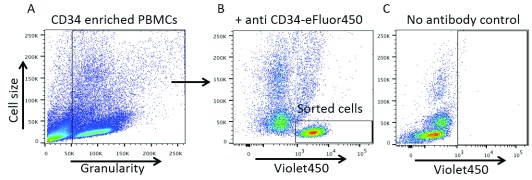
Anti-CD34 antibody staining identifies a clear cell population in CD34 enriched cells by flow cytometry. ( **A**) Healthy cells only were gated to analyse fluorescence. ( **B**) A clear cell population (black rectangle) of small cells was positive for the Violet450 fluorophore as determined by the no antibody control ( **C**) where that same cell population is shifted to the left of the X-axis.

In human stem transplantation therapy, successful transplantation correlates with high number of injected CD34 positive cells ( Zaucha *et al.*, 2001). To produce a population of human cells that would mimic a human HSC graft, we used the CD34 marker to purify HSC from whole blood. The method, summarised in Figure 1 and detailed in Supplementary File 1, consists of multiple steps in order to ensure robust and consistent purification of CD34 cells. Whole blood was collected from healthy volunteers and immediately processed to extract neutrophils and PBMCs. The PBMC fraction was then enriched for CD34 cells using a positive selection magnetic column. These cells were then further labelled with a human anti-CD34eFluor450 antibody and sorted using FACS. Only cells positive for the eFluor450 fluorophore were sorted; therefore ensuring a pure CD34 cell population ( Figure 2).

**Figure 3.**
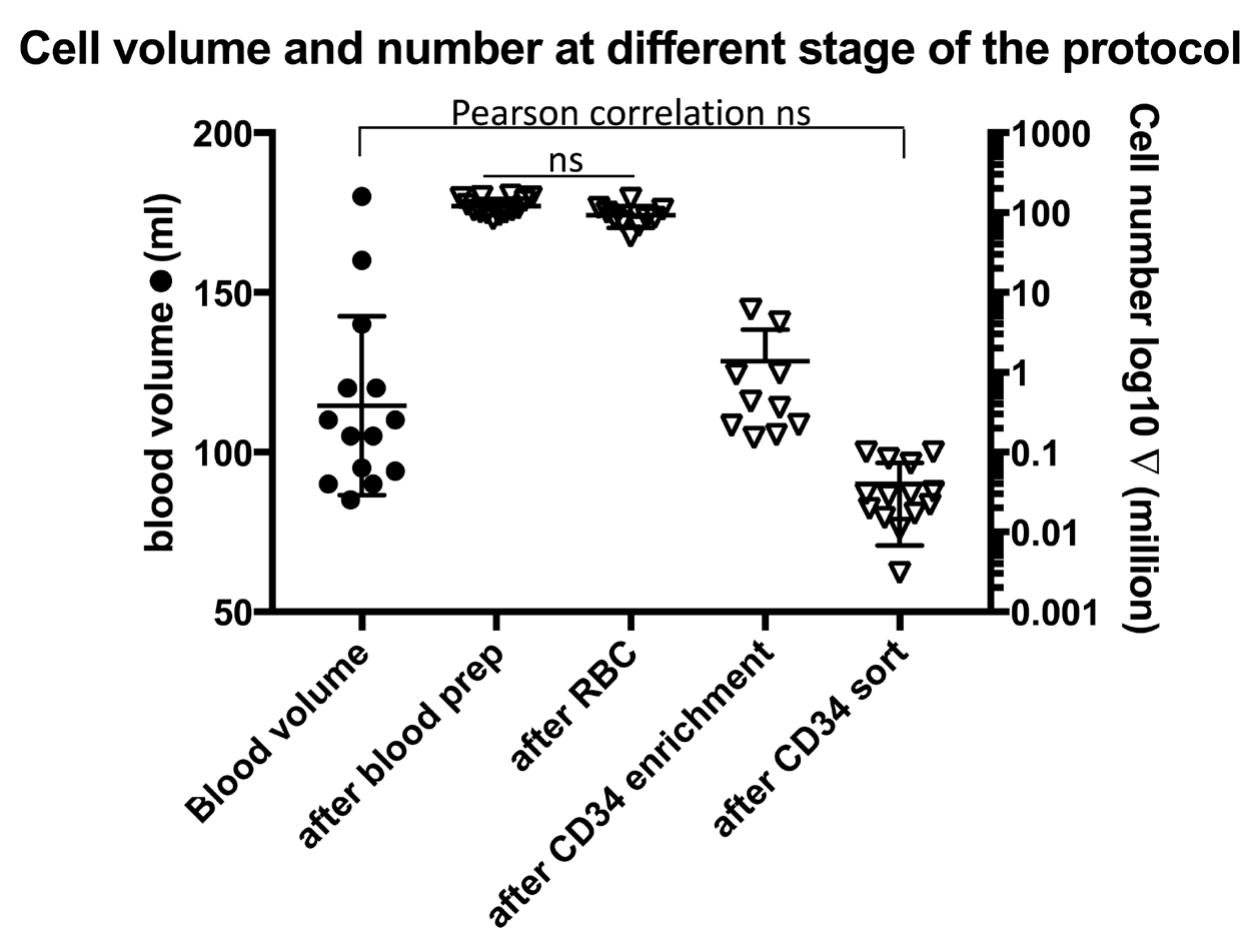
CD34 cells represent a small fraction of PBMCs. Left scale represent the blood volume taken per donors. Paired T-test was used to analyse statistical significance between ‘after blood prep group’ and ‘after red blood cell (RBC) lysis group’ (n=10). Paired Pearson correlation analysis was performed between the blood volume taken and the final number of sorted CD34 cells and no correlation was found (p= 0.115, n=14, Pearson r=0.441).

***CD34 cells represent a small fraction of PBMCs.*** During each experiment, cells were counted at each specific point of the protocol and expected ranges of cells have also been noted on the protocol. The volume of blood taken varied between 50ml and 180ml (left axis Figure 3). Cell number was counted on a haemocytometer after each important step of the protocol. Number of cells after PBMCs isolation varied between 83 and 162.5 millions, and after red blood cell (RBC) lysis numbers ranged from 50.6 and 149.6 millions. Of note, our results show no significant difference in PBMC number after RBC lysis ( Figure 3, n=14, Paired T-test). After CD34 enrichment, cells were counted again and varied between 0.152 and 6.15 millions. Finally, after cell sorting, we recorded a range of pure CD34 cells between 3000 and 100,000. As expected, as the purity of CD34 cells increased, the cell number dramatically decreased ( Figure 3). On average, CD34 positive cells represented 0.033% of total PBMCs recovered from the cell preparation (n=10). Moreover, paired Pearson correlation analysis was performed between the blood volume taken and the final number of sorted CD34 cells and no correlation was found (p= 0.115, n=14, Pearson r=0.441). This may be due to the high variability in the pool of CD34 cells between donors.

**Figure 4.**
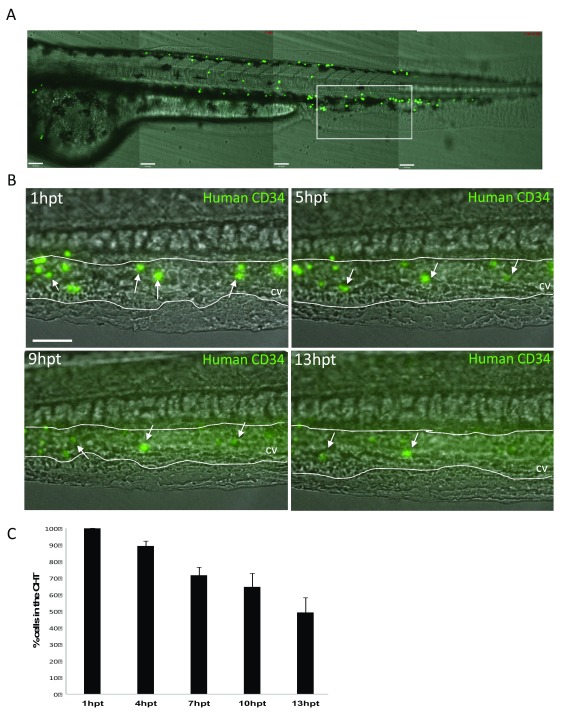
Injected human CD34 cells quickly appear in the zebrafish Caudal Haematopoietic Tissue (CHT). ( **A**) Stitched Z-stack of whole Zebrafish larvae trunk highlighting the CHT (white rectangle). ( **B**) Representative Z-Stack images of fluorescein labelled human CD34 cells present at the CHT at 1hour post transplantation (hpt), 5hpt, 9hpt and 13hpt. Scale bar=80μm. ( **C**) Quantification of the decreasing total number of CD34 human cells in the CHT in embryos injected with fluorescein labelled human CD34 cells (n=5). First image of the CHT taken at 1hpt representing 100% of cells within the CHT and quantified following single emrbyos at 5hpt, 9hpt and 13hpt.

***Injected human CD34 cells adhere to the zebrafish CHT.*** Purified human CD34 cells were labelled with fluorescein and injected into the blood circulation by targeting the Duct of Cuvier in 52hpf zebrafish larvae ( Figure 1). We first observed that human CD34 cells are visible in the zebrafish CHT immediately after injection ( Figure 4A) where they appeared to adhere to the endothelial wall of the blood vessels forming the CHT. Subsequently, instead of being washed away from the CHT by the blood flow, human CD34 cells were seen to roll and tether along the caudal vein. This behaviour has previously been reported for endogenous zebrafish HSCs, known to emerge from the ventral wall of aorta to join the circulation and roll along the endothelium of the caudal vein to reach their haematopoietic niche ( Gering & Patient, 2005; Kissa & Herbomel, 2010; Tamplin *et al.*, 2015). We quantified how many cells stayed in the CHT by counting the number of GFP positive cells at 1dpf, 5hpt, 9hpt and 13hpt. The highest number of human CD34 cells initially adhered to the wall of the caudal vein in the CHT was seen within one hour after injection ( Figure 4B). We counted how many GFP positive cells were present in the CHT at 5hpt, 9hpt and 13hpt following single embryos (n=5) ( Figure 4C). We plotted the percentage of cells still present compared to the initial number (100%) and showed that only 50% of these initial cells are still present in the CHT at 13hpt ( Figure 4C).

**Figure 5.**
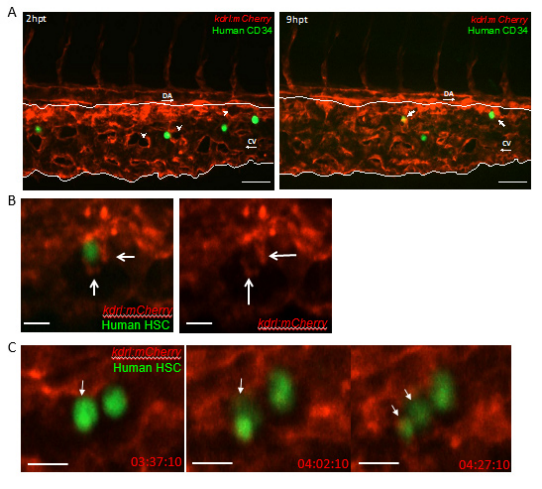
Human CD34 cells interact with zebrafish endothelial cells. Spinning disk confocal stills of timelapses zebrafish Caudal Haematopoietic Tissue (white lines) from the endothelial cell (red) reporter line *Tg(kdrl:mCherry)* zebrafish larvae transplanted with human CD34 cells (green). ( **A**) At 2hpt, human CD34 cells are still in the vessels and empty perivascular pockets (white arrowheads) do not contain human CD34 cells. At 9hpt, human CD34 cells co-localised with endothelial cells and human CD34 cells appears inside the perivascular pockets (white arrows). DA: Dorsal aorta, CV: Caudal vein. Scale bar= 50μm. ( **B**) High magnification of a human CD34 cell being ‘cuddled’ by surrounding endothelial cells, note the endothelial cell protrusion acting like arms (white arrows). Single Z plane from a Z-stack. Scale bar= 50μm. N=1 ( **C**) Division of a human CD34 cells (white arrow) within a perivascular pocket of a 56hpf embryos, with hours post transplantation (hpt) displayed. Note the equal distribution of fluorescence between the two daughter cells in the last frame. N=1. Single Z plane from a Z-stack. Scale bar=10μm.

***Human CD34 cells lodge in zebrafish haematopoietic niches.*** It is known that zebrafish and mouse HSCs, once adhered to the caudal vein, enter perivascular pockets proximally to the caudal vein ( Figure 5A, white arrowheads) ( Kiel *et al.*, 2005; Nombela-Arrieta *et al.*, 2013; Tamplin *et al.*, 2015). To assess whether human CD34 cells interact with zebrafish endothelial cells, we transplanted CD34 human cells into the *Tg(kdrl:mCherry)* reporter line labelling endothelial cells in red ( Chi *et al.*, 2008). Spinning disk confocal images focusing on the zebrafish CHT showed that at 2 hours post transplantation (hpt) human CD34 cells are still located in the caudal vein ( Figure 5A). At 9hpt, we observed co-localisation of human CD34 cells with endothelial cells, with some human CD34 cells already inside the perivascular pockets ( Figure 5A). Once inside the perivascular pocket, we observed interactions with endothelial cells within the pocket, this process has already been termed ‘cuddling’ when imaging endogenous zebrafish HSCs: endothelial cells surround and embrace the incoming stem cell ( Tamplin *et al.*, 2015). Clear extensions, positive for the endothelial cell marker, were observed surrounding a human CD34 cell from within the perivascular pocket ( Figure 5B, Supplementary Movie 1). Moreover, we imaged an instance where human CD34 cells divide within the zebrafish haematopoietic niche ( Figure 5C, Supplementary Movie 2). These observations demonstrated that these human CD34 cells have engrafted into the zebrafish CHT, therefore confirming that the zebrafish native CHT provides a human-compatible environment to allow human cells to engraft.

FACS output files for Figure 2 Click here for additional data file. Copyright: © 2018 Hamilton N et al. 2018 Data associated with the article are available under the terms of the Creative Commons Zero "No rights reserved" data waiver (CC0 1.0 Public domain dedication).

Raw values file for Figure 3 Click here for additional data file. Copyright: © 2018 Hamilton N et al. 2018 Data associated with the article are available under the terms of the Creative Commons Zero "No rights reserved" data waiver (CC0 1.0 Public domain dedication).

Raw values file and image file for Figure 4 Click here for additional data file. Copyright: © 2018 Hamilton N et al. 2018 Data associated with the article are available under the terms of the Creative Commons Zero "No rights reserved" data waiver (CC0 1.0 Public domain dedication).

Raw image file for Figure 5. Images should be opened with Velocity software Click here for additional data file. Copyright: © 2018 Hamilton N et al. 2018 Data associated with the article are available under the terms of the Creative Commons Zero "No rights reserved" data waiver (CC0 1.0 Public domain dedication).

## Discussion

Our protocol of human HSCs transplantation in zebrafish resulted in similar events observed in the numerous studies on zebrafish haematopoiesis. Zebrafish haematopoiesis has been well described and it is known that endogenous HSCs emerge from the ventral wall of aorta to join the circulation at around 36 hours post fertilisation ( Gering & Patient, 2005; Kissa & Herbomel, 2010). HSCs roll along the endothelium of the caudal vein in the zebrafish CHT, where they exit the blood vessel to reach perivascular pockets of endothelial cells ( Gering & Patient, 2005; Kissa & Herbomel, 2010; Tamplin *et al.*, 2015). This haematopoietic niche protects the stem cell and allows it to divide and colonise other haematopoietic tissues. The well-described engraftment process of zebrafish HSCs in the CHT has provided us with key cell engraftment behaviour to look for.

Indeed, we have observed that human CD34 cells home into the zebrafish CHT, where they engage with endothelial cells. By using high resolution confocal microscopy on transplanted zebrafish from the *Tg(kdrl:mCherry)* endothelial cells reporter line, we observed that human CD34 cells exit the caudal vein to reach perivascular pockets of endothelial cells within 9 hours after injection. Once in perivascular pockets, human CD34+ cells are ‘cuddled’ by endothelial cells and even divide, processes already described for zebrafish endogenous stem cells.

*Future validation assays:* Injections of 20 to 50 labelled human CD34+ cells in the circulation of zebrafish larvae is enough to observe interactions with endothelial cells in perivascular pockets and subsequent division within the zebrafish CHT. To validate the extent to which this interaction can represent engraftment, stem cell colonisation of the definitive haematopoietic organs and further cell differentiation must be studied. Stem cell colonisation of the definitive haematopoietic organs starts from day 5, the thymus is colonised by HSCs and later on the kidney marrow, both organs which will contribute to the development of the adaptive immune system ( Kissa *et al.*, 2008). Future validation assays looking at stem cell migration to the thymus and kidneys would be useful to confirm the extent of engraftment. Once engrafted, HSCs will produce lineage-committed progenitors that will give rise to blood cells, including immune cells. To further validate the model, populations of human blood cells present in adult zebrafish could be measured. It was shown that enriched human CD34 cells injected into zebrafish larvae resulted in the presence of myeloid lineage human cells only ( Staal *et al.*, 2016). Our protocol using pure CD34 cells may provide a better graft and differentiate into multiple lineages. Engraftment of human HSCs could potentially affect the development of endogenous zebrafish HSC and the morphology of the CHT, which could both influence the longevity of the graft. This could be assessed by using zebrafish specific HSCs markers, such as *runx1*, and CHT morphology markers such as the *tg(kdrl:mCherry)* line ( Chi *et al.*, 2008). Moreover, the innate immune response to the presence of human CD34 cells in the CHT should be studied, as this could uncover potential limitations to engraftment success due to removal of transplanted cells by macrophages and neutrophils.

*Limitations of our current protocol:* Currently our protocol detailed a method for purifying CD34 cells from whole blood, known to contain a small number of circulating HSCs. Indeed we have shown that blood samples used for cell preparation contained on average 0.03% of CD34 cells. Our current protocol allows the transplantation of 20–50 cells per fish from a pool of minimum 10×10 ^3^ cells, allowing transplantation of maximum 100 fish. Although this would be sufficient to assess efficiency and viability of a single graft, it would be limiting to use as a high-throughput assay. This protocol could be scaled up using automated injection to transplant thousands of zebrafish larvae to perform drug screens, or even to apply different HSC markers to study engraftment properties of human HSCs. However, a larger pool of HSCs will be required. Parada-Kusz *et al.* described a high-throughput transplantation assay of murine CD34 cells from bone marrow in zebrafish ( Parada-Kusz *et al.*, 2017). Bone marrow from crushed femurs contains considerably more CD34 cells than whole blood, but this source cannot be use for human donors. To obtain a larger pool of HSCs, cord blood and enriched whole blood after cytokine G-CSF treatment would be suitable sources. These samples can be obtained through necessary ethical approvals and will provide enough graft material to scale up our protocol and perform high-throughput assays.

It is important to note that the transplanted embryos were raised at normal zebrafish temperature of 28 degree Celsius (°C). Raising the temperature might improve survival of human CD34 cells, as it will provide a more ‘human like’ environment, without affecting the embryos development. Indeed, raising zebrafish embryos at a higher temperature of 36 °C after xenotransplantation of human tumour cells showed no effect on embryo survival and improved survival of the human cells ( Cabezas-Sainz *et al.*, 2018).

*Translatability:* Alongside offering a new model to continue research on stem cell transplantation, our zebrafish assay offers a scientific advantage that could revolutionise how stem cell graft are being tested before transplantation. Currently, most patients in need of transplantation receive a graft that has been cryopreserved and stored at suprafreezing temperatures ( Stockschläder *et al.*, 1997). Although these grafts are being tested for viability using the Trypan Blue staining, there is currently no assay quick enough to test for efficacy of the graft to engraft ( Fleming & Hubel, 2006). Our data show the potential of human CD34+ cells to colonise the haematopoietic niche in zebrafish, engraft and proliferate within 9 hours after injection. Therefore, our zebrafish assay detailed in this study could provide a quick, cheap and efficient assay to test graft efficiency and even viability in less than 12h.

*Transferability:* Our zebrafish system to study stem cell transplantation research will also advance the 3Rs components with a major impact on animal welfare. The replacement of humanised mice by humanised zebrafish larvae will represent a giant step for the 3Rs, allowing zebrafish embryos to be a host for human stem cells without any myeloablative procedures. The refinement of the harmful procedures without the need of myeloablation, by using zebrafish before the development of adaptive immunity, represents a powerful alternative to mice. Moreover, using a transparent organism before the onset of independent feeding to visualise stem cell engraftment will allow selection of engrafted animal within 12h. Zebrafish are widely used and for communities without zebrafish facilities, a small zebrafish system to host the few adults needed for this experiment is easy and cheap to start. These critical advantages of this assay using zebrafish as a model system will, we hope, increase the chances of wide uptake of this system.

## Data availability

The data referenced by this article are under copyright with the following copyright statement: Copyright: © 2018 Hamilton N et al.

Data associated with the article are available under the terms of the Creative Commons Zero "No rights reserved" data waiver (CC0 1.0 Public domain dedication). 

**Dataset 1: FACS output files for Figure 2.** DOI: 10.5256/f1000research.14507.d200844 ( Hamilton *et al.*, 2018a)

**Dataset 2: Raw values file for Figure 3.** DOI: 10.5256/f1000research.14507.d200845 ( Hamilton *et al.*, 2018b)

**Dataset 3: Raw values file and image file for Figure 4.** DOI: 10.5256/f1000research.14507.d200847 ( Hamilton *et al.*, 2018c)

**Dataset 4: Raw image file for Figure 5.** Images should be opened with Velocity software. DOI: 10.5256/f1000research.14507.d200848 ( Hamilton *et al.*, 2018d)

## Supplementary material

Supplementary File 1: Full protocol for transplantation of human HSCs into zebrafish.

.

Supplementary File 2: NC3R ARRIVE checklist.

.

Supplementary Movie 1: Human stem cell being cuddled by zebrafish endothelial cells.

.

Supplementary Movie 2: Human stem cell dividing within zebrafish perivascular pocket.
