# Supplementary material for: A method for transplantation of human HSCs into zebrafish, to replace humanised murine transplantation models
Source: F1000Res. 2018 Dec 23;7:594. Originally published 2018 May 15. [Version 2] doi: 10.12688/f1000research.14507.2 (PMC6008850; doi:10.12688/f1000research.14507.2)
Supplement: Supplementary file 5 [file f1000research-7-19274-s0004.tgz › 7e35e51b-d1c2-46fb-8d93-c80ff864396e.pdf]

A human HSCs transplantation method into zebrafish to replace humanised murine transplantation models.

Noémie Hamilton[1,2], Ian Sabroe[2], Stephen A Renshaw[1,2]

1. The Bateson Centre, University of Sheffield, Firth Court D31, Sheffield S10 2PT

2.Department of Infection, Immunity and Cardiovascular Disease, University of Sheffield, Sheffield, United Kingdom.

Supplementary File 1

Protocol

Adult Zebrafish from the *tg(kdrl:mCherry)* line were bred and viable embryos were sorted in groups of 50 and raised in 10ml of 1x E3 fish medium at 28 degree until 48 hours post fertilisation (hpf). The duration of each step of the protocol should be used as a timeline to plan experiments accordingly. Transplantation into the zebrafish larvae was performed when the larvae have reached 52hpf.

E3 medium 60x: 5mM NaCl, 0.17mM KCl, 0.33mM $CaCl_2$, 0.33mM $MgSO_4$, 10^{-5} % Methylene Blue

Protocol Visual Summary

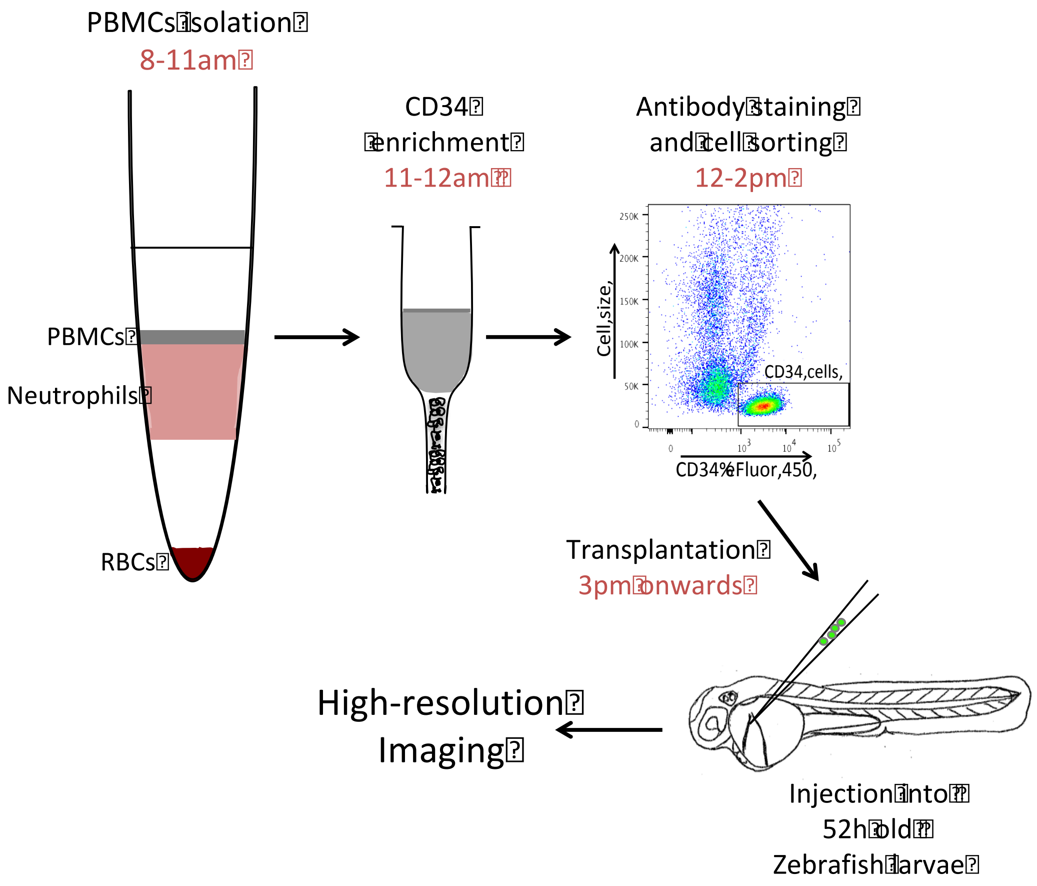

I. Peripheral blood mononuclear cells isolation (8 →11am)

A. Reagents and solutions

1. Reagents

Sodium citrate 3.8% w/v, 10 X 10.5ml ampoules (Martindale Pharmaceuticals), Sodium bicarbonate (cat. VX 25080060 Fisher), HBSS Ca2+, Mg2+ & NACO3 free (Gibco), Dextran (cat. D5251 Sigma), Percoll (cat. P1644 Sigma), Water, sterile (cat. UKF7114 Baxter), 0.9% saline (w/v) (Pharmacy), Butterfly needles, Parafilm, 50ml Syringes, 50ml Falcon Tubes, 15ml Falcon Tubes, Stripettes, sterile Pasteur pipettes.

2. Solutions

6% Dextran: To make preferably the day before the experiment. Place 3g of Dextran in a 50ml tube and add 40ml of saline. Vortex to mix and put in water bath at 37˚C to dissolve. Make up to 50ml with saline and store at 4˚C.
90% Percoll: Place 9ml of Percoll in a 15ml tube and make up to 10ml with saline. Store at 4˚C. Aliquot out the stock of Percoll and store at -20˚C.
1X HBSS: Add 5ml of 10x HBSS and 45ml of sterile water to a 50ml Falcon.

B. Blood collection

1. Prior to collect blood, remove the Dextran and Percoll solution from the fridge and leave to reach room temperature in the hood. Minimum of 50ml blood volume recommended to obtain enough CD34 cells to transplant.

    a) Range of volume in blood taken: 50ml-180ml

2. Place 4ml of sodium citrate into a 50ml tube and make sure it reaches room temperature being adding the blood.

3. Take blood from human volunteers using a butterfly needle into a 50ml syringe.

    a) Note: It is very important to be gentle with the blood samples as activation of neutrophils could disrupt the final gradient and contaminate the PBMC layer.

4. Immediately when the syringe is full, let blood cascade down the side of the 50ml tube with Sodium Citrate. Close the tube with the lid, gently roll and invert the tube to mix blood.

5. Add parafilm around the lid and spin at 1200rpm for 20 minutes at room temperature.

6. Aspirate the clear orange top layer which is the platelet rich plasma without disturbing or touching the bottom layer and transfer into a 50ml Falcon tube.

7. Put the platelet rich plasma into a 50ml tube and spin at 2000rpm for 20 minutes

8. Collect supernatant into a 50ml Falcon tube to make platelet poor plasma (PPP) and discard the pellet.

C. Dextran separation

1. Add gently 6ml of 6% dextran solution to the cell pellet and immediately make up to 50ml with saline.

2. Roll the tube slowly to resuspend the cells.

   a) Note: Use a plastic pipette to remove droplets in the lid and remove bubbles on the surface at all time to avoid neutrophil activation

3. Loosen the lid and leave to sediment for 30 minutes or until there is a clear interface.

4. After dextran sedimentation, remove the clear cell rich upper layer and place in a 50ml centrifuge tube.

5. Spin at 1000rpm for 6 minutes to pellet the cells.

D. Percoll Gradient

1. Prepare Percoll gradient solutions

   a) Note: This has to be accurate to work well and ensure there are no bubbles into the solution once the gradient is assembled.

   - Make up 2ml of upper 42% Percoll in a labelled 15ml falcon tube using: 0.84ml of 90% Percoll + 1.16ml of PPP
   - Make up 2ml of lower 51% Percoll in a labelled 15ml falcon tube using: 1.02ml of 90% Percoll + 0.98ml of PPP.

2. Make the gradient using the overlaying method. Using a sterile Pasteur pipette carefully overlay the 42% Percoll solution on top of the 51% first layer in the 15ml falcon tube.

   a) Note: It is best to tilt the tube when doing this and let the 42% solution gently land on top of the 52% solution.

3. Keep the gradient upright in a corner of the hood where nobody will move the tube.

4. When the spin is finished, carefully discard as much of the liquid as possible without losing cells.

5. Gently resuspend the cells in 2ml of PPP using a sterile Pasteur pipette.

6. Using the same sterile Pasteur pipette, overlay the cells on top of the gradient by gently lowering the cell solution onto the gradient by tilting the tube.

7. Spin at 1100rpm for 11 minutes with brake set to zero.

8. Gently remove the tube from the centrifuge, there should be clearly separated multiple layers of PBMCs, neutrophils and Red Blood Cells as shown below.

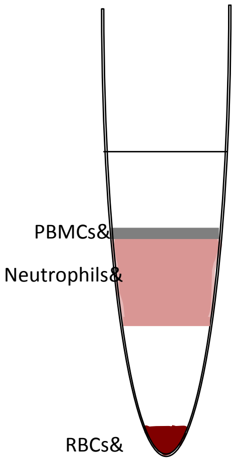

Cell distribution after Percoll gradient

E. PMBCs selection and counting

1. Make up to 50ml of 1x HBSS.

2. Insert a sterile Pasteur pipette just above the PBMCs layer and hoover the layer.

   a) Note: Remove the neutrophil layer using the same technique if needed for another assay.

3. Transfer into the HBSS 50ml tube. Count cells using a haemocytometer.

   a) Range of cell count at this stage: 83→162.5millions

4. Spin at 1500rpm for 6 minutes.

II. CD34 enrichment (11 →12am)

A. Reagents

PBSE (PBS-2mM EDTA), CD34 MicroBead kit (Miltenyl Biotech, Bergisch Gladbach, Germany), LS columns (Miltenyl Biotech, Bergisch Gladbach, Germany), Magnetic field support and magnetic separator for LS columns (Miltenyl Biotech, Bergisch Gladbach, Germany), MACS buffer (PBS, EDTA 2mM, 0.5% Bovine Serum Albumin), 50ml Falcon Tubes, 15ml Falcon Tubes, Stripettes, sterile Pasteur pipettes.

B. Red Blood Cell (RBC) lysis

1. Resuspend PBMC pellet in 10ml of PBSE.

2. Spin at 1500rpm for 5 minutes.

3. Resuspend pellet in 10ml of 1x RBC buffer kept at room temperature and incubate 2 minutes. Add 5µl to a haemocytometer to determine the efficiency of the RBC lysis

    a) ***Note: If RBC are still visible under the microscope, leave the cells in the RBC lysis buffer for longer as RBC will saturate the magnetic column.***

4. Spin at 1500rpm for 5 minutes.

5. Resuspend in PBSE

6. Add 5µl to a haemocytometer to determine the efficiency of the RBC lysis and count PMBCs.

    a) ***Range of cell count at this stage: 50.6 →149.6 millions***

C. CD34 Enrichment with Myltenyi kit

1. Spin at 1500rpm for 5 minutes.

2. Resuspend in 300µl fridge cold MACS buffer (not ice cold). Collect 10µl in a 1.5ml Eppendorf tube, add 1ml of MACS buffer and keep on ice as unlabelled PBMC sample for FACS

3. Add 100µl Fc Block + 100µl CD34 beads from CD34 MicroBead Kit.

4. Incubate for 30min in fridge

5. Wash with 10ml of MACS

6. Spin at 1500rpm for 5 minutes.

7. Resuspend in 3ml of cold MACS- keep samples on ice

8. Position an LS column onto the magnetic adaptor and equilibrate with 3ml MACS

9. Load column with sample and rinse the sample falcon tube with 0.5ml of MACS buffer to collect all the cells and add to the column. Let the sample go through the column by gravity and collect the flow through (FT) in a 50ml falcon tube.

10. Wash column with 3x3ml MACS

11. Remove column from magnetic separator and place over a 15ml falcon. Add 3ml of MACS into the column and elute the cells by using the plunger provided with the column.

12. Repeat using 3ml of MACS buffer

13. Spin down the collected FT, resuspend in 1ml of MACS buffer and pass through the same column again.

   a) ***Note: We found that too many CD34 cells were still present in the FT and doing another round of magnetic selection minimised cell loss.***

14. Combine both elutes together and count cells.

   a) ***Range of cell count at this stage: 0.152 →6.15 millions***

III. Antibody staining and cell sorting (12-2pm)

A. Reagents

Human anti-CD34 eFLuor450 antibody (eBioscience), MACS buffer, Eppendorf tubes.

B. Antibody staining

1. Spin at 1500rpm for 5 minutes.

2. Remove supernatant down to 1ml, resuspend cells and transfer to a 1.5ml Eppendorf tube.

   a) ***Note: This is to ensure that the small cell pellet is not lost as it is usually loosely attached to the 15ml falcon tube. Transferring the last 1ml of liquid ensure there is no cell loss.***

3. Spin the Eppendorf tube at 1500rpm for 5 minutes in a tabletop centrifuge.

4. For the first time the experiment is performed, an unstained sample of CD34 enriched cells is necessary to set up the gating on the FACS machine. Therefore resuspend the cells in 180µl. Place 90µl in a 1.5ml Eppendorf tube to use as non-labelled CD34 cells and perform the antibody staining as describe below on the other 90µl. For the next experiments, to have as many CD34 cells as possible to transplant, use the previously kept unlabelled PBMCs to set the gates. Otherwise, resuspend CD34 enriched cells in 90µl of cold MACS buffer and add 5µl of CD34 antibody.

5. Incubate in the fridge for 10 minutes (Do not exceed 30 minutes of incubation)

6. Add 1ml of MACS buffer and spin at 1500rpm for 5 minutes.

7. Remove all the supernatant and wash cells with another 1ml of MACS buffer

8. Spin at 1500rpm for 5 minutes.

9. Remove all the supernatant and resuspend in 1ml of MACS buffer. Store on ice before proceeding to FACS.

C. Cell sorting

We have used a FACsARIA machine to sort CD34 positive cells. Gating was made using unlabelled samples and positive cells were sorted into cold MACS buffer.

a) Range of cell sorted: 3000 → 100000

IV. Transplantation and Imaging (3pm onwards)

A. Reagents and solutions

CFSE 10mM stock solution in DMSO (eBioscience), Polyvinylpyrrolidone (PVP Sigma PVP40), Phenol Red (Sigma), E3 Fish water, Tricaine MS222 (Sigma), 2% Agarose in E3 plates. Low melting point agarose (Sigma), E3 zebrafish media, Tricaine MS222 (Sigma), borosilicate glass capillaries for needles (World Precision Instrument, TW100-4), 35mm glass coverslip bottom petri dish (Matek), graticule, mineral oil.

<u>Making Needles</u>: Needles were made from borosilicate glass capillaries using a Needle Puller (Sutter Instrument Model P-1000), using the following settings: Heat= 390, Pull=220, Vel=200, Ramp=382, Time=200, Pressure=500.

<u>10% PVP</u>: Measure 2g of PVP and add 20ml of Phenol red. Autoclave and use at desired concentration.

B. Labelling with fluorescein

1. Add CFSE to the sorted cells at 1:10000 dilution and incubate for 10 minutes.

2. Spin at 1500rpm for 5 minutes.

3. Remove supernatant and resuspend cells in 1% PVP at a minimum of 10000cells/μl.

C. Transplantation

1. Load injection needle with resuspended cells and snap the end of the needle with sharp thin tweezers. Calibrate the needle to dispense 1nl of volume into a drop of mineral oil on a graticule.

2. Sedate *Tg(kdrl:mCherry)* larvae with Tricaine and align up to 20 larvae on an agarose plate, head to the left.

3. Insert the needle into a large flat vein on top of the yolk sac, also called duct of Cuvier and inject 2nl of cell suspension. A Jove detailed video for injection into the Duct of Cuvier is available at Bernard et al, 2012.

4. Once the 20 larvae have been injected, transfer to a petri dish with fresh E3 media and keep at 28 degrees. Repeat until you have ran out of cells to inject. On average, a maximum of 100 larvae can be injected with the amount of cells collected.

5. Sedate injected animals and select larvae with Fluorescein labelled human cells in their caudal hematopoietic tissue (CHT) using the blue laser under a fluorescent scope. If injections were performed in the Duct of Cuvier, all animals will have cells in their CHT. Select animals with more than 10 cells in the CHT to increase chances to visualise translocation into perivascular pockets.

D. Mounting and Imaging

1. Make 0.8% low melting point (LMP) agarose in E3 and keep melted agarose at 37 degree in a heat block.

2. Embed positive larvae on their side in 0.8% LMP agarose on a 35mm glass coverslip bottom petri dish to image using a Spinning Disk confocal microscope, objective x40 to provide high-resolution images of human CD34 cells interacting with zebrafish endothelial cells. Minimum time of imaging was 8 hours to see human cells interacting with endothelial cells. Larvae in agarose were sedated with a lethal dose of Tricaine and disposed in bleach.
