# Supplementary material for: A method for transplantation of human HSCs into zebrafish, to replace humanised murine transplantation models
Source: F1000Res. 2018 Dec 23;7:594. Originally published 2018 May 15. [Version 2] doi: 10.12688/f1000research.14507.2 (PMC6008850; doi:10.12688/f1000research.14507.2)
Supplement: Peer review history

# Peer review: Referee response for version 1

**DOI:** 10.5256/f1000research.15792.r34964

**Published:** 2018-06-19

**Authors:** Stuart M. Allan, Siobhan Crilly

**Affiliations:** 1 University of Manchester, Manchester, UK; 2 Faculty of Medical and Human Sciences, The University of Manchester, Manchester, UK

The methods article from Hamilton *et al *provides a strong rationale for an improved and accessible model for HSC engraftment in a larval zebrafish. Humanised mouse models require severe procedure methodology and limitations in visualising the engraftment process essential for understanding human mechanisms and zebrafish offer an alternative, complementary model in which to do so.

 The methodology provided is detailed enough to be translated and repeated accurately. As the authors highlight, this model has potential for be up-scaled for high-throughput methodology for drug screening and offers a necessary complementary model to reduce the number of rodents needed for pre-clinical study. Further investigation in this model would prove interesting to see how engrafted HSCs develop into various lineages and employing the porous nature of the zebrafish embryo to investigate drug treatment to encourage engraftment/proliferation/lineage development.

 Points to consider for revision:

1. When isolating the CD34+ cells using both magnetic beads and antibodies, do the authors remove these markers before transplantation?
2. Have the authors investigated an innate immune cellular response to the engraftment of human cells?
3. How do cells react to the changes in temperature (37 -28 degrees) for zebrafish incubation? Have the authors experimented using different incubation temperatures for fish comparable to some tumour lines?
4. How does the engraftment impact the development of native zebrafish HSCs (from 30hpf) and production of haematopoietic markers?
5. How does the changing productivity of the CHT tissue (in the developing organism) affect the longevity of the engraftment?
6. What happens to the model after the development of the mature adaptive immune system in the zebrafish after 2 weeks – is this the end of the experiment, and engraftment and lineage cannot be investigated further than this?
7. How many of the cells that ‘lodged’ into the tissue went on to proliferate at later timepoints 2-13hpt?
8. Please ensure full details of statistical analyses that were performed are provided in the methods and also that the results of these analyses are described in the main text. Also the figure legends should include details of the statistical comparisons made with any significant results being indicated on the relevant figures.

 

 Minor corrections in the manuscript:

- ‘Transplanted’ should be ‘transplantation’ on page 4 left column line 5
- Can the authors label figure 4 as done in figure 5, the dorsal aorta, caudal vein and stem cell tissue.
- In the ‘future validation assay’ section the authors say kidney bone marrow instead of kidney marrow
- What time point are the images in 5C taken?

**Are a suitable application and appropriate end-users identified?**

 Yes

**If applicable, is the statistical analysis and its interpretation appropriate?**

 Partly

**Are the 3Rs implications of the work described accurately?**

 Yes

We have read this submission. We believe that we have an appropriate level of expertise to confirm that it is of an acceptable scientific standard.

# Response

**Published:** 2018-12-18

**Authors:** Noemie Hamilton

**Affiliations:** University of Sheffield, UK

We thank Prof Allan and Miss Crilly for their positive comments. We have uploaded an improved version of the manuscript including all the minor corrections they suggested. Their recommended revisions have also been added to the manuscript and detailed below points by points:

1. We have added this to the methods. The antibody and the beads were not been removed, however we have been advised by the company selling the CD34 MicroBead kit that the beads naturally detach within 24 hours. 
2. We thank you for this suggestion and we hope that the innate immune cellular response to the engraftment of transplanted cells can be part of another study. We have added your suggestion as part of the ‘future validation assays’ of our discussion.
3. A very recent study (Cabezas-Sainz et al., 2018) has explored the effect of raising the incubation temperature of zebrafish to accommodate for human cells and we have added to the  ‘Limitations’ section of our discussion. 
4. We have not investigated whether the engraftment of new cells had an impact on the development of endogenous zebrafish HSCs, nor the integrity of the CHT (point 5). However, we have added this to our discussion as an important future validation assay.
5. Included in point 4 above.
6. What happens to the model after the development of the mature adaptive immune system in the zebrafish after 2 weeks has already been mentioned in the validation assay part of the discussion. We agree that this is a crucial experiment to perform to investigate the presence of different lineages emerging from the engrafted human HSCs. However, our current experiments end at 5dpf  and raising chimera animals past this age is not within the scope of this study.
7. We have added the full details of statistical analyses to our figure legends, results sections and have uploaded updated figures.  

# Peer review: Referee response for version 1

**DOI:** 10.5256/f1000research.15792.r34036

**Published:** 2018-06-04

**Authors:** Owen J. Tamplin

**Affiliations:** 1 Department of Pharmacology, University of Illinois at Chicago, Chicago, IL, USA

This method article by Hamilton et al. describes in extensive detail the procedures involved in transplantation of human CD34+ hematopoietic stem/progenitor cells (HSPCs) directly into the circulation of 2 day-old zebrafish embryos. Although there have been studies that transplanted mouse ^1^ and human ^2^ HSPCs into zebrafish embryos as a model for homing and lodgement, as the authors discuss, this article provides the technical details necessary to successfully reproduce these experiments. The authors explain clearly how this approach can reduce the number of animal models needed in research (i.e., the 3Rs). Zebrafish embryos as transplant recipients can be collected in the hundreds each week, without the need to sacrifice adult animals. The efficiency of the approach is shown by the small number of CD34+ HSPCs that are needed per recipient, about 50 per embryo, compared to about 10,000 per recipient mouse. The authors perform time-lapse live imaging that reveals transplanted human HSPCs are “cuddled” by zebrafish endothelial cells in the niche, a dynamic cellular behavior that is observed during endogenous HSPC lodgement ^3^. Overall, the data presented in this method article is of high quality, is sufficient to reproduce the technique, and provides a strong rationale for the 3Rs. However, as other similar studies have been performed ^2^, it would be helpful if the authors could further extend their results to highlight improvements to the method.

 Some suggestions for revisions are listed below:

1. What are the different options for dyes that could be used to label donor cells? Has toxicity been assessed in dosage curves? Have alternatives been explored? How does PKH26 ^2^compare to fluorescein (this study)?
2. How long do the donor cells survive in the transplant recipient? Staal et al. track the cells until 6 dpf, so 4 days after transplant at 2 dpf. How long were recipients followed in these experiments?
3. How does temperature affect survival of donor cells, given the different optimal temperatures between human and zebrafish (i.e., 37C vs 28C).
4. From the existing data collected for the study, what percentage of lodged HSPCs are found in pockets?
5. Have other injection methods been tried? RO injection in embryos is also possible, and may prove easier and more efficient than injection into the Duct of Cuvier.
6. How were transplants controlled? Is there a way to distinguish between lodged viable cells vs stuck debris? If sorted adult zebrafish HSPCs were transplanted in similar numbers (e.g., kidney cd41:gfp low cells), would the lodged number of cells be comparable to human CD34+ cells?
7. Staal et al. tested chemokine responses *ex vivo*—could the authors inject human cytokines into zebrafish recipient embryos to test their effects *in vivo*? For example, would the transplant results change in the presence of human G-CSF?

 

 Possible additional “Discussion” points:

1. How could the method be scaled up for higher throughput studies (e.g., automated injection)?
2. What are the limitations of the system compared with mice? How could the non-isogenic background of zebrafish impact a study, compared to using isogenic mouse recipients?
3. Is ‘engraftment’ appropriate terminology, given that the cells can only be tracked short-term? Would ‘lodgement’ be a better description of the processed that is modeled?
4. Replacing the adult mouse with the zebrafish embryo as a transplant recipient for human HSPCs switches the system from an adult bone marrow niche to a developmental “fetal-like” niche (i.e., CHT). How should this be considered when interpreting the results?

 

 Minor points related to the manuscript:

1. Could the authors provide higher resolution images in figure 4A-B?
2. The absolute numbers should also be shown in Figure 4C. What is the number inside and outside of the CHT at 1 hpt? Does 100% mean there are no circulating cells in the embryo at 1 hpt? If there are 50% fewer cells in the CHT 12 hours later, where are the cells going? Are they migrating to a different tissue or dying?
3. How long is the image in Figure 4A taken after injection? Text says 100% after injection are in the caudal vein, but the image shows cells around the embryo.
4. Are the images in Figure 5 maximum projections or single slices? Single Z planes more clearly show the endothelial projections. In Figure 5B, it should be clear that the second frame is only endothelial cells. Single channels could be shown together with the merge.
5. This text on page 3 is not clear: “These immunodepleted mice must be grown to adulthood in order to assess engraftment success, usually performed shortly after birth.” Does this refer to a neonatal transplant?
6. How were the numbers collected for this statement on page 3: “In 2015, 25 publications used immunocompromised mice for HSC transplant studies, representing around 1000 mice each year worldwide – all undergoing severe procedures over a long period of time.” Is there a reference that could be cited?

**Are a suitable application and appropriate end-users identified?**

 Yes

**If applicable, is the statistical analysis and its interpretation appropriate?**

 Yes

**Are the 3Rs implications of the work described accurately?**

 Yes

I have read this submission. I believe that I have an appropriate level of expertise to confirm that it is of an acceptable scientific standard.

## References

[1] Parada-KuszMMClatworthyAHagedornEJet al.: Generation of Mouse-Zebrafish Hematopoietic Tissue Chimeric Embryos for Hematopoiesis and Host-Pathogen Interaction Studies. *bioRxiv*.2017;10.1242/dmm.034876PMC626281630266803

[2] StaalFJSpainkHPFibbeWE: Visualizing Human Hematopoietic Stem Cell Trafficking In Vivo Using a Zebrafish Xenograft Model. *Stem Cells Dev*.2016;25(4) : 10.1089/scd.2015.0195 360-5 10.1089/scd.2015.0195 26650921

[3] TamplinOJDurandEMCarrLAChildsSJHagedornEJLiPYzaguirreADSpeckNAZonLI: Hematopoietic stem cell arrival triggers dynamic remodeling of the perivascular niche. *Cell*.2015;160(1-2) : 10.1016/j.cell.2014.12.032 241-52 10.1016/j.cell.2014.12.032 25594182PMC4346256

# Response

**Published:** 2018-12-18

**Authors:** Noemie Hamilton

**Affiliations:** University of Sheffield, UK

We thank Professor Owen Tamplin for his positive review and encouraging comments for revisions. We have uploaded a revised manuscripts and updated figures addressing all of his minor comments. We have responded to his suggestions and additional discussion points below:

Some suggestions for revisions are listed below:

1. We have not tested different dyes for this study and we have stuck to using a green dye to go alongside the red Tg(kdrl:mcherry) line.
2. What happens to the model after 5dpf is a crucial experiment to validate the assay and ensure the human cells have truly engrafted. This is already part of the discussion, mentioning other studies looking at the different lineages emerging from the engrafted human HSCs. However, our current experiments end at 5dpf  and raising chimera animals past this age would require an amendment of our project licence which is not within the scope of this study.
3. A very recent study (Cabezas-Sainz et al., 2018) has explored the effect of raising the incubation temperature of zebrafish to accommodate for human cells and we have added to the  ‘Limitations’ section of our discussion. 
4. We have not tried other routes of injection and we are grateful for the RO injection suggestion which will be tested.
5. We have not transplanted zebrafish CD41 cells and we would expect them to engraft better than human cells.
6. This is a great suggestion and this is how we hope this model will be used in the future: To find new molecules that could improve engraftment success.

 Possible additional “Discussion” points:

1. We have added the automated injection to our ‘Limitations of our current protocol’ section to go alongside sourcing CD34 from different sources to scale up the pt
2. The limitations of using a non-isogenic recipient and an ‘foetal-like niche’ are very valid points. We hope that our zebrafish embryonic model will be used instead of mice to optimise protocols initially. We fully expect that mice will still be used to corroborate results from zebrafish studies before being used in patients.
3. We agree that ‘lodge’ would be a more suitable description for our model until we show long term engraftment of human cells. This was replaced in the result section. 
4. Replacing the adult mouse with the zebrafish embryo as a transplant recipient for human HSPCs switches the system from an adult bone marrow niche to a developmental “fetal-like” niche (i.e., CHT). How should this be considered when interpreting the results?

 Minor points related to the manuscript:

- 1+2+3: Figure 4: we apologise for the confusion in the quantification from Figure 4C. We have changed the text and figure legend to be more consistent and emphasise that the absolute number of cells was the number of cells lodged within the CHT at 1hpt. We subsequently quantified the number of cells at 5hpt, 9hpt and 13hpt and plotted them in percentage. We do not find GFP positive cells in any other tissues after transplantation, so we can conclude that the cells initially visualised in the CHT could be debris or healthy cells that have died and be removed by innate immune cells.
- Figure 5b and 5C are all single plane, we have specified this in the figure legend
- We have modified the text on page 3 to make sure readers do not think we are referring to neonatal transplant. ‘These immunodepleted mice must be grown to adulthood in order to assess engraftment success. They live their entire lives undergoing severe procedures with high maintenance requirements, since they need to be homed in sterile rooms and fed sterile food to avoid fatal infection due to defective immunity.’
- Publications using immune compromised mice were counted using a PubMed search, reading articles and averaging how many mice were used per study. We have added this to the text to make it clearer.
